# Patient-reported outcomes in clinical trials leading to breast cancer drug approvals: are we making them count?

**DOI:** 10.1007/s10549-026-08051-y

**Published:** 2026-08-10

**Authors:** Samuel Margolis, Andre Naguib Guirguis, Catherine Lee, Saiya Smith, Alexis LeVee, Marla D. Lipsyc-Sharf, Aditya Bardia, Mina S. Sedrak

**Affiliations:** 1 Department of Medicine, University of California, Los Angeles, CA USA; 2 David Geffen School of Medicine at UCLA, 650 Charles Young Drive South, Room A2-125 CHS, Los Angeles, CA 90095-6900 USA

**Keywords:** Patient-reported outcomes, Breast cancer, Clinical trials, Drug approval, Quality of life, Regulatory science, Toxicity, Tolerability

## Abstract

**Purpose:**

Patient-reported outcomes (PROs) inform benefit-risk tradeoffs in breast cancer treatment, yet reporting quality across registration trials remains incompletely characterized.

**Methods:**

We conducted a scoping review of PRO reporting in randomized trials supporting FDA breast cancer drug approvals (January 2011–December 2024), identified via the FDA Oncology Approval Notifications database. Associated manuscripts were retrieved through systematic PubMed and EBSCO searches using trial-specific NCT identifiers. We assessed PRO endpoint prespecification status, instruments used, PRO publication delay (defined as the interval in months between the primary trial manuscript and corresponding PRO-dedicated manuscript publication date), and missing data reporting across eligible primary trial, PRO-dedicated, and subpopulation-specific PRO manuscripts.

**Results:**

We identified 34 FDA breast cancer drug approvals supported by 36 unique randomized trials, yielding 45 eligible manuscripts. No trial designated PROs as a primary or co-primary endpoint; nine (25%) did not explicitly prespecify them. Median PRO publication delay was 18 months, with eight trials experiencing a delay of two or more years. Missing data handling methods were described in 33% of manuscripts and reasons for missingness in 11%.

**Conclusion:**

In this scoping review, PROs were commonly collected but inconsistently prespecified, often delayed, and incompletely reported. Registration trials should strengthen PRO prespecification, reduce publication delay, and adhere to missing data reporting standards.

**Clinical trial number:**

Not applicable.

**Supplementary Information:**

The online version contains supplementary material available at 10.1007/s10549-026-08051-y.

## Introduction

Patient-reported outcomes (PROs) capture data on symptoms, functional limitations, and quality of life directly from patients [1, 2]. In breast cancer, treatment decisions often require balancing survival benefits against risks of treatment-related adverse effects on health and quality of life; accurate assessment of this benefit-risk tradeoff depends on the availability of high-quality PRO data from clinical trials [3, 4]. However, despite increasing recognition of the importance of PROs, reporting practices remain variable and are not consistently aligned with established guidance [5, 6]. In breast cancer registration trials specifically, the extent to which PROs are prioritized with respect to endpoint prespecification, timeliness of dissemination, and missing data reporting remains incompletely characterized [7, 8].

We performed a scoping review to evaluate these domains across trials supporting U.S. Food and Drug Administration (FDA) breast cancer drug approvals.

## Methods

### Article search and selection

We identified eligible FDA breast cancer drug approvals by reviewing the FDA Oncology (Cancer)/Hematologic Malignancies Approval Notifications database from January 2011 through December 2024, extracting the National Clinical Trial (NCT) identifier for each to locate the supporting record on ClinicalTrials.gov. We systematically searched PubMed and EBSCO for associated manuscripts, combining PRO-related terms with the trial-specific NCT number. Eligible manuscripts represented one of three types: a primary trial manuscript, a PRO-dedicated manuscript, or a subpopulation-specific PRO analysis. Conference abstracts, non-English manuscripts, and manuscripts combining results from more than one trial were excluded. Because approvals rather than unique drugs were used to define trial eligibility, agents approved for more than one indication or line of therapy could contribute more than one approval to our search (Supplementary Table S1).

### Data extraction and analysis

For each eligible trial, we abstracted PRO endpoint classification, sample size, and the number and type of PRO instruments used.

PRO endpoint prespecification status was classified as secondary, exploratory, both, or not explicitly prespecified, based on endpoint labeling in the trial protocol and/or primary manuscript. We reviewed each trial’s ClinicalTrials.gov record and trial protocol to confirm whether PRO collection was planned.

Timeliness of PRO dissemination was assessed by calculating PRO publication delay, defined as the interval in months between the primary trial manuscript and the corresponding PRO-dedicated manuscript publication date; for trials with more than one PRO-dedicated manuscript, the earliest was used for trial-level summaries.

Missing data reporting was assessed by reviewing each manuscript and supplementary files for any mention of: missing data, quantitative reporting of the extent of missingness, a description of the method used to handle missing data, or whether reasons for missingness were provided.

Categorical variables were summarized as counts and proportions. Continuous variables were summarized with descriptive statistics.

## Results

Our search identified 34 FDA breast cancer drug approvals between January 2011 and December 2024, supported by 36 unique randomized clinical trials. These 34 approvals corresponded to 19 unique drugs, as several agents were approved for more than one indication or line of therapy during the study period (Supplementary Table S1). Forty-five manuscripts reporting PRO data met eligibility criteria, including 26 (57%) PRO-dedicated manuscripts, seven (16%) subpopulation-specific PRO analyses, and 12 (27%) primary trial manuscripts containing PRO results.

Among the 36 trials, 31 (86%) were phase III and five (14%) were phase II. No trials included PROs as primary or co-primary endpoints. PROs were included as secondary endpoints in 23 trials (64%), as exploratory endpoints in three trials (8%), and as both secondary and exploratory endpoints in one trial (3%). Nine trials (25%) did not explicitly list PROs as prespecified endpoints. The median number of PRO instruments per trial was two (range 0–4). PRO prespecification and instrument selection did not differ appreciably by treatment setting (metastatic/palliative vs. curative/adjuvant/neoadjuvant) or by drug class (Supplementary Table S1). The EORTC QLQ-C30, EQ-5D, and EORTC QLQ-BR23 were the most frequently used instruments across trials; FACIT-based and symptom-specific measures were used less consistently (Table 1).

**Table 1:** PRO instruments used across trials (*N* = 36) ^a^

Instrument	Trials, *n* (%)
EORTC QLQ-C30	21 (58)
EQ-5D, any version^b^	13 (36)
EORTC QLQ-BR23	12 (33)
FACT-B	5 (14)
BPI-SF or Modified BPI-SF	4 (11)
FACIT-Fatigue	2 (6)
EORTC QLQ-BR45	1 (3)
FACT-ES	1 (3)
PRO-CTCAE	1 (3)
SID Satisfaction Questionnaire	1 (3)

^a^Totals exceed 36; most trials used multiple instruments. No specific PRO instrument was identified in 7 trials (19%). Median instruments per trial, 2 (range, 0–4)

^b^EQ-5D-5 L in 8 trials; EQ-5D-3 L in 1 trial; version not specified in 4 trials

**Fig. 1:**
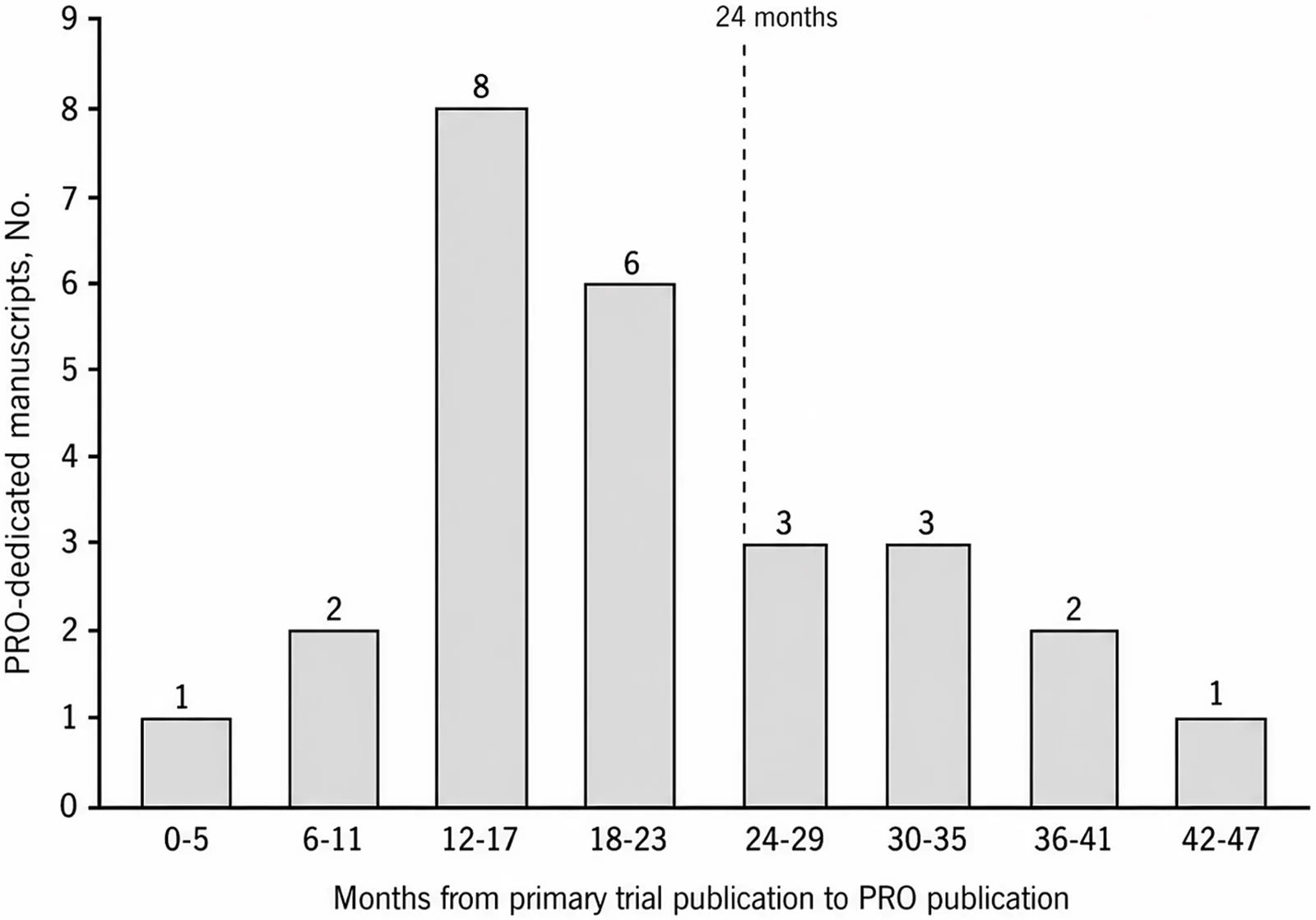
Time from primary trial publication to PRO-dedicated publication. Distribution of publication delay among 26 PRO-dedicated manuscripts identified across FDA breast cancer registration trials. Publication delay was defined as the number of months between publication of the primary trial manuscript and publication of the corresponding PRO-dedicated manuscript

All 36 trials had at least one manuscript reporting PRO data. Among the 26 PRO-dedicated manuscripts, the median time from primary trial manuscript to PRO publication was 18 months (mean 20.8 months; range 0–44 months) (Fig. 1). Eight trials experienced a PRO publication delay of two or more years. PRO publication delay did not show a consistent trend across the study period.

Fifteen of 45 manuscripts (33%) described a method for handling missing PRO data, and five (11%) reported reasons for missingness, including participant reporting burden, disease progression, and treatment discontinuation.

## Discussion

In this scoping review we evaluated PRO endpoint prespecification, timeliness of dissemination, and missing data reporting in FDA registration trials supporting breast cancer drug approvals. One in four trials did not prespecify PROs as endpoints, the breast cancer–specific EORTC QLQ-BR23 and FACT-B were used in only 12 and 5 trials, respectively, and missing data characterization was limited. These findings reflect a unifying theme that PRO data is under collected and inconsistently translated into usable clinical information.

PRO endpoint prespecification was absent in one-quarter of trials, a finding consistent with prior oncology reporting audits; these gaps motivated the development of CONSORT-PRO and SPIRIT-PRO reporting guidelines [5, 6, 9]. Without explicit prespecification, PRO results cannot be interpreted as hypothesis-driven evidence and are highly susceptible to selective post hoc framing. This is corroborated by the limited use of disease-specific surveys, because general surveys do not fully capture disease-relevant declines in QoL [9, 10–11].

PRO median publication time was 18 months, with eight trials experiencing a delay of two or more years. This is consistent with broader concerns about timely reporting in registration trials [12]. Contributing factors may include the lower priority assigned to PRO endpoints relative to survival outcomes, publication strategy, and sponsor incentives. Formal FDA guidance requiring prespecification of PRO endpoints and defined timelines for PRO-dedicated publication, paralleling existing requirements for efficacy endpoints, may help standardize and accelerate PRO reporting in future breast cancer registration trials. PRO data that have not yet been published may not inform treatment decisions in the years following drug approval [12]. 

Missing data reporting was inadequate in most manuscripts; only one-third described a method for handling missing PRO data, and only five (11%) reported reasons for missingness. Transparent missing data reporting is particularly important because missingness in PRO data is frequently non-random: patients who discontinue treatment due to disease progression or worsening symptoms are least likely to complete assessments, and their exclusion can bias estimates in ways that are undetectable without such reporting, thus violating SISAQOL reporting standards [7].

This review has several limitations. First, the modest number of trials (*n* = 36) limited our ability to formally test associations between trial-level characteristics and PRO reporting outcomes; we examined these relationships descriptively to avoid overinterpretation of underpowered comparisons. Second, our search was restricted to PubMed and EBSCO and may not have captured all eligible manuscripts. Third, because our analysis relied on published literature, PRO data collected but not yet published could not be captured, potentially underestimating true PRO reporting. Fourth, this review focused specifically on breast cancer registration trials, and results may not generalize to other tumor types or non-registrational trials.

Our findings indicate that PRO endpoint prespecification was inconsistent, with dissemination delays, and incomplete missing data reporting. PROs are central to interpreting treatment tradeoffs in breast cancer, where symptom burden, functional status, and quality of life influence treatment selection alongside efficacy outcomes [1, 3, 4]. Future registration trial reporting practices should strengthen PRO endpoint prespecification, establish clearer expectations for timeliness of PRO dissemination, and adhere to reporting standards for missing data handling so that PRO data can be more useful for clinical decision making, regulatory interpretation, and guideline development [9–13]. 

## Supplementary Information

Below is the link to the electronic supplementary material.

Supplementary Material 1

## Data Availability

No datasets were generated or analysed during the current study.
