# Supplementary material for: Patient-reported outcomes in clinical trials leading to breast cancer drug approvals: are we making them count?
Source: Breast Cancer Res Treat. 2026 Aug 10;218(3):35. doi: 10.1007/s10549-026-08051-y (PMC13457446; doi:10.1007/s10549-026-08051-y)
Supplement: Supplementary file 1 [file 10549_2026_8051_MOESM1_ESM.docx]

**Supplementary Table S1a. Trial and clinical characteristics of randomized clinical trials supporting FDA breast cancer drug approvals (2011–2024).**

| **Approval date** | **Trial** | **NCT identifier** | **Intervention** | **Drug class** | **Indication (setting)** | **Phase** | **Primary efficacy endpoint** | **Reference** |
| --- | --- | --- | --- | --- | --- | --- | --- | --- |
| Nov 16, 2023 | CAPItello-291 | NCT04305496 | capivasertib + fulvestrant | AKT inhibitor | HR+/HER2−; metastatic (2L) | III | PFS | Turner et al. N Engl J Med. 2023. |
| Feb 3, 2023 | TROPiCS-02 | NCT03901339 | sacituzumab govitecan-hziy | ADC | HR+/HER2−; metastatic (3L) | III | PFS | Rugo et al. J Clin Oncol. 2022. |
| Jan 27, 2023 | EMERALD | NCT03778931 | elacestrant | SERD (oral) | HR+/HER2−; metastatic (2L) | III | PFS | Bidard et al. J Clin Oncol. 2022. |
| Aug 5, 2022 | DESTINY-Breast04 | NCT03734029 | fam-trastuzumab deruxtecan-nxki | ADC | HER2-low; metastatic (2L) | III | PFS | Modi et al. N Engl J Med. 2022. |
| May 4, 2022 | DESTINY-Breast03 | NCT03529110 | fam-trastuzumab deruxtecan-nxki | ADC | HER2+; metastatic (2L) | III | PFS | Cortés et al. N Engl J Med. 2022. |
| Mar 11, 2022 | OlympiA | NCT02032823 | olaparib | PARP inhibitor | HER2−, BRCA+; adjuvant | III | iDFS | Tutt et al. N Engl J Med. 2021. |
| Oct 12, 2021 | monarchE | NCT03155997 | abemaciclib + endocrine therapy | CDK4/6 inhibitor | HR+/HER2−; adjuvant | III | iDFS | Johnston et al. J Clin Oncol. 2020. |
| Jul 26, 2021 | KEYNOTE-522 | NCT03036488 | pembrolizumab | Immune checkpoint inhibitor | TNBC; neoadjuvant/adjuvant | III | pCR & EFS | Schmid et al. N Engl J Med. 2022. |
| Apr 7, 2021 | ASCENT | NCT02574455 | sacituzumab govitecan | ADC | TNBC; metastatic (3L) | III | PFS | Bardia et al. N Engl J Med. 2021. |
| Dec 16, 2020 | SOPHIA | NCT02492711 | margetuximab-cmkb | HER2 mAb | HER2+; metastatic (3L) | III | PFS & OS | Rugo et al. JAMA Oncol. 2021. |
| Nov 13, 2020 | KEYNOTE-355 | NCT02819518 | pembrolizumab | Immune checkpoint inhibitor | TNBC; metastatic (1L) | III | PFS & OS | Cortés et al. Lancet. 2020. |
| Jun 29, 2020 | FeDeriCa | NCT03493854 | pertuzumab + trastuzumab + hyaluronidase (SC) | HER2 mAb | HER2+; neoadjuvant/adjuvant | III | PK (serum Ctrough)† | Tan et al. Lancet Oncol. 2020. |
| Apr 22, 2020 | IMMU-132-01 | NCT01631552 | sacituzumab govitecan-hziy | ADC | TNBC; metastatic (3L) | II | ORR† | Ocean et al. Cancer. 2017. |
| Apr 17, 2020 | HER2CLIMB | NCT02614794 | tucatinib + trastuzumab + capecitabine | HER2 TKI | HER2+; metastatic (2L) | III | PFS | Murthy et al. N Engl J Med. 2019. |
| Feb 25, 2020 | NALA | NCT01808573 | neratinib + capecitabine | HER2 TKI | HER2+; metastatic (3L) | III | PFS | Saura et al. J Clin Oncol. 2020. |
| Dec 20, 2019 | DESTINY-Breast01 | NCT03248492 | fam-trastuzumab deruxtecan-nxki | ADC | HER2+; metastatic (3L) | II | ORR | Modi et al. N Engl J Med. 2019. |
| May 24, 2019 | SOLAR-1 | NCT02437318 | alpelisib | PI3K inhibitor | HR+/HER2−; metastatic (2L) | III | PFS | André et al. N Engl J Med. 2019. |
| May 3, 2019 | KATHERINE | NCT01772472 | ado-trastuzumab emtansine | ADC | HER2+; adjuvant | III | iDFS | von Minckwitz et al. N Engl J Med. 2018. |
| Mar 8, 2019 | IMpassion130 | NCT02425891 | atezolizumab | Immune checkpoint inhibitor | TNBC; metastatic (1L) | III | PFS & OS | Schmid et al. N Engl J Med. 2018. |
| Feb 28, 2019 | HannaH | NCT00950300 | trastuzumab + hyaluronidase (SC) | HER2 mAb | HER2+; neoadjuvant/adjuvant | III | pCR & serum Ctrough† | Ismael et al. Lancet Oncol. 2012. |
| Feb 28, 2019 | SafeHER | NCT01566721 | trastuzumab + hyaluronidase (SC) | HER2 mAb | HER2+; neoadjuvant/adjuvant | III | Safety (AE incidence)† | Gligorov et al. Eur J Cancer. 2017. |
| Oct 16, 2018 | EMBRACA | NCT01945775 | talazoparib | PARP inhibitor | HER2−, BRCA+; metastatic (2L) | III | PFS | Litton et al. N Engl J Med. 2018. |
| Jul 18, 2018 | MONALEESA-7 | NCT02278120 | ribociclib | CDK4/6 inhibitor | HR+/HER2−; metastatic (1L) | III | PFS | Tripathy et al. Lancet Oncol. 2018. |
| Feb 26, 2018 | MONARCH 3 | NCT02246621 | abemaciclib | CDK4/6 inhibitor | HR+/HER2−; metastatic (1L) | III | PFS | Goetz et al. J Clin Oncol. 2017. |
| Jan 12, 2018 | OlympiAD | NCT02000622 | olaparib | PARP inhibitor | HER2−, BRCA+; metastatic (2L) | III | PFS | Robson et al. N Engl J Med. 2017. |
| Dec 20, 2017 | APHINITY | NCT01358877 | pertuzumab | HER2 mAb | HER2+; adjuvant | III | iDFS | von Minckwitz et al. N Engl J Med. 2017. |
| Sep 28, 2017 | MONARCH 2 | NCT02107703 | abemaciclib | CDK4/6 inhibitor | HR+/HER2−; metastatic (2L) | III | PFS | Sledge et al. J Clin Oncol. 2017. |
| Sep 28, 2017 | MONARCH 1 | NCT02102490 | abemaciclib | CDK4/6 inhibitor | HR+/HER2−; metastatic (3L) | II | ORR | Dickler et al. Clin Cancer Res. 2017. |
| Jul 17, 2017 | ExteNET | NCT00878709 | neratinib | HER2 TKI | HER2+; extended adjuvant | III | iDFS | Chan et al. Lancet Oncol. 2016. |
| Mar 31, 2017 | PALOMA-2 | NCT01740427 | palbociclib | CDK4/6 inhibitor | HR+/HER2−; metastatic (1L) | III | PFS | Finn et al. N Engl J Med. 2016. |
| Feb 19, 2016 | PALOMA-3 | NCT01942135 | palbociclib | CDK4/6 inhibitor | HR+/HER2−; metastatic (2L) | III | PFS | Turner et al. N Engl J Med. 2015. |
| Feb 3, 2015 | PALOMA-1 | NCT00721409 | palbociclib | CDK4/6 inhibitor | HR+/HER2−; metastatic (1L) | II | PFS† | Finn et al. Lancet Oncol. 2014. |
| Sep 30, 2013 | NeoSphere | NCT00545688 | pertuzumab | HER2 mAb | HER2+; neoadjuvant | II | pCR | Gianni et al. Lancet Oncol. 2011. |
| Feb 22, 2013 | EMILIA | NCT00829166 | ado-trastuzumab emtansine | ADC | HER2+; metastatic (3L) | III | PFS & OS | Verma et al. N Engl J Med. 2012. |
| Jul 20, 2012 | BOLERO-2 | NCT00863655 | everolimus | mTOR inhibitor | HR+/HER2−; metastatic (3L) | III | PFS | Baselga et al. N Engl J Med. 2012. |
| Jun 8, 2012 | CLEOPATRA | NCT00567190 | pertuzumab | HER2 mAb | HER2+; metastatic (1L) | III | PFS | Baselga et al. N Engl J Med. 2012. |

**Supplementary Table S1b. Patient-reported outcome reporting for the same 36 trials, in the same order.**

| **Trial** | **PRO endpoint status** | **PRO instrument(s)** | **Primary publication year** | **PRO publication year** | **PRO delay, mo** |
| --- | --- | --- | --- | --- | --- |
| CAPItello-291 | Secondary | EORTC QLQ-C30, EORTC QLQ-BR23 | 2023 | NR | NR |
| TROPiCS-02 | Secondary | EORTC QLQ-C30, EQ-5D-5L, PRO-CTCAE | 2022 | 2024 | 20 |
| EMERALD | Secondary | EORTC QLQ-C30 | 2022 | NR | NR |
| DESTINY-Breast04 | Not prespecified | None identified | 2022 | NR | NR |
| DESTINY-Breast03 | Secondary | EORTC QLQ-C30, EORTC QLQ-BR45, EQ-5D-5L | 2022 | 2023 | 13 |
| OlympiA | Secondary | EORTC QLQ-C30, FACIT-Fatigue | 2021 | 2024 | 31 |
| monarchE | Secondary | FACT-B, FACT-ES, FACIT-Fatigue, EQ-5D-5L | 2020 | 2022 | 18 |
| KEYNOTE-522 | Secondary | EORTC QLQ-C30, EORTC QLQ-BR23 | 2022 | NR | NR |
| ASCENT | Secondary | EORTC QLQ-C30 | 2021 | 2022 | 17 |
| SOPHIA | Not prespecified | None identified | 2021 | NR | NR |
| KEYNOTE-355 | Secondary | EORTC QLQ-C30, EORTC QLQ-BR23, EQ-5D, version not specified | 2020 | 2023 | 36 |
| FeDeriCa | Not prespecified | None identified | 2020 | NR | NR |
| IMMU-132-01 | Not prespecified | None identified | 2017 | NR | NR |
| HER2CLIMB | Exploratory | EQ-5D-5L | 2019 | 2021 | 18 |
| NALA | Secondary | EORTC QLQ-C30, EORTC QLQ-BR23, EQ-5D-5L | 2020 | 2021 | 9 |
| DESTINY-Breast01 | Not prespecified | None identified | 2019 | NR | NR |
| SOLAR-1 | Secondary & exploratory | EORTC QLQ-C30, BPI-SF | 2019 | 2021 | 22 |
| KATHERINE | Secondary | EORTC QLQ-C30, EORTC QLQ-BR23 | 2018 | 2020 | 16 |
| IMpassion130 | Secondary | EORTC QLQ-C30, EORTC QLQ-BR23 | 2018 | 2020 | 16 |
| HannaH | Not prespecified | None identified | 2012 | NR | NR |
| SafeHER | Not prespecified | SID Satisfaction Questionnaire | 2017 | NR | NR |
| EMBRACA | Exploratory | EORTC QLQ-C30, EORTC QLQ-BR23 | 2018 | 2018 | 0 |
| MONALEESA-7 | Secondary | EORTC QLQ-C30, EQ-5D-5L | 2018 | 2020 | 26 |
| MONARCH 3 | Secondary | EORTC QLQ-C30, EORTC QLQ-BR23, EQ-5D-5L | 2017 | 2020 | 32 |
| OlympiAD | Secondary | EORTC QLQ-C30 | 2017 | 2019 | 24 |
| APHINITY | Secondary | EORTC QLQ-C30, EORTC QLQ-BR23, EQ-5D-3L | 2017 | 2021 | 44 |
| MONARCH 2 | Secondary | EORTC QLQ-C30, EORTC QLQ-BR23, EQ-5D-5L, Modified BPI-SF | 2017 | 2019 | 28 |
| MONARCH 1 | Not prespecified | EORTC QLQ-C30, Modified BPI-SF | 2017 | NR | NR |
| ExteNET | Exploratory | FACT-B, EQ-5D, version not specified | 2016 | 2019 | 35 |
| PALOMA-2 | Secondary | FACT-B, EQ-5D, version not specified | 2016 | 2018 | 14 |
| PALOMA-3 | Secondary | EORTC QLQ-C30, EORTC QLQ-BR23, EQ-5D, version not specified | 2015 | 2016 | 8 |
| PALOMA-1 | Secondary | Modified BPI-SF | 2014 | 2016 | 14 |
| NeoSphere | Not prespecified | None identified | 2011 | NR | NR |
| EMILIA | Secondary | FACT-B | 2012 | 2013 | 12 |
| BOLERO-2 | Secondary | EORTC QLQ-C30, EORTC QLQ-BR23 | 2012 | 2013 | 13 |
| CLEOPATRA | Secondary | FACT-B | 2012 | 2013 | 18 |

Abbreviations: 1L/2L/3L, first/second/third-line therapy; ADC, antibody-drug conjugate; AE, adverse event; BRCA, BRCA1/2 mutation; CDK4/6, cyclin-dependent kinase 4/6; EFS, event-free survival; HER2, human epidermal growth factor receptor 2; HR, hormone receptor; iDFS, invasive disease-free survival; mAb, monoclonal antibody; NCT, National Clinical Trial identifier; NR, not reported (no PRO-dedicated manuscript identified); ORR, objective response rate; OS, overall survival; pCR, pathological complete response; PARP, poly(ADP-ribose) polymerase; PFS, progression-free survival; PI3K, phosphoinositide 3-kinase; PK, pharmacokinetic; PRO, patient-reported outcome; SC, subcutaneous; SERD, selective estrogen receptor degrader; TKI, tyrosine kinase inhibitor; TNBC, triple-negative breast cancer.

Both tables are ordered by FDA approval date (most recent first). Treatment setting reflects the approval indication: metastatic settings are palliative, and adjuvant/neoadjuvant settings are curative. Trials were deduplicated by NCT identifier; one trastuzumab biosimilar (Herzuma, approved December 14, 2018) had no independent registration trial and was excluded, and one duplicate MONALEESA-7 record was removed (38 − 1 − 1 = 36 trials). The 34 approvals corresponded to 19 unique drugs. PRO publication delay (Table S1b) is the interval in months between the primary trial manuscript and the earliest PRO-dedicated manuscript.

† The registered primary outcome was a pharmacokinetic or safety measure (e.g., subcutaneous-formulation bridging or single-arm studies); the efficacy endpoint shown is the key secondary or co-primary endpoint.
